# Unlocking Therapeutic Potential: Comprehensive Extraction, Profiling, and Pharmacological Evaluation of Bioactive Compounds from *Eclipta alba* (L.) Hassk. for Dermatological Applications

**DOI:** 10.3390/plants13010033

**Published:** 2023-12-21

**Authors:** Hla Myo, Desy Liana, Anuchit Phanumartwiwath

**Affiliations:** College of Public Health Sciences, Chulalongkorn University, Bangkok 10330, Thailand; hlamyo.dr1996@gmail.com (H.M.); desyliana13@gmail.com (D.L.)

**Keywords:** *Eclipta alba*, *Eclipta prostrata*, extraction, identification, phytochemicals, separation

## Abstract

Herbal medicine has been studied as an alternate approach to modern medicine as it is more cost-effective and accessible via natural sources. *Eclipta alba* (*E. alba*, L.) Hassk. is a weed plant abundantly distributed throughout different regions of the world and contains abundant bioactive compounds used for various skin conditions. In this review, we aimed to gather information from the literature about the extraction, separation, and identification of these bioactive compounds and their potential in skin diseases. Relevant studies published before August 2023 were identified and selected from electronic databases, including Scopus, SciFinder, ScienceDirect, Google Scholar, and Wiley Library, using the following keywords: *Eclipta alba*, *Eclipta prostrata*, phytochemicals, extraction, separation, isolation, identification, characterization, pharmacological activity, and skin conditions. Up-to-date extraction, separation, and identification methods of bioactive compounds from *E. alba* and their skin-related pharmacological activities are discussed in this review. As there are limitations regarding extraction, separation, and identification methods, and in-depth mechanistic and human studies of the skin-related pharmacological activities of bioactive compounds, these gaps are areas for future research to expand our understanding and broaden the potential applications of this medicinal weed plant, including the development of cosmeceutical and skincare products, anti-inflammatory agents, and formulations for dermatological treatments.

## 1. Introduction

Herbal medicine has been increasingly used as a cost-effective and natural alternative to modern medicine, with a focus on avoiding the possible side effects of modern medicine and combating diseases more naturally. Moreover, people can inform themselves about herbal therapy and its potential advantages via social media and the internet. In terms of evidence-based medicine, the use of herbal medicines is still an issue of ongoing debate [1]. Despite reliability and quality control issues related to herbal medicines, the exploration of natural sources for bioactive compounds has gained significant momentum in the pursuit of innovative treatments for various health conditions [2].

**Figure 1:**
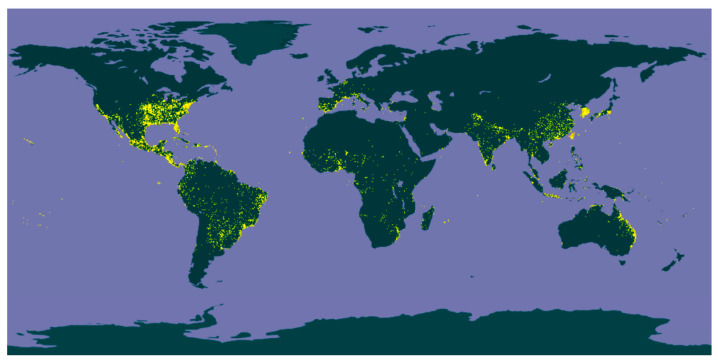
Updated distribution map of *Eclipta alba* (L.) Hassk. [9] (Source: Global Biodiversity Information Facility Secretariat (GBIF), https://www.gbif.org/species/5384950 (accessed on 13 December 2023)).

*Eclipta alba* (*E. alba*, L.) Hassk., also called *Eclipta prostrata* (L.) Linn., a member of the Asteraceae family, is a naturally occurring weed plant. It is called false daisy in English [3]. This herbaceous plant, which has tiny branches and white flower clusters, thrives as an annual in damp environments. *E. alba* is abundantly distributed throughout different regions of the world, as shown in Figure 1, including Africa, America, India, China, Myanmar, and Thailand, and it has been regarded as a weed plant in many countries [4]. *E. alba* contains rich phytochemical components constituting the various parts of the plant [5], including coumestan derivatives, steroids, triterpenoids, steroidal and triterpene saponins, flavonoids, and phenols [5,6,7]. Various parts of the plant have been applied in traditional Asian medicine for gastrointestinal disorders, skin conditions, high blood pressure, hepatic disorders, and wounds. In Myanmar, it has been extensively used for promoting hair growth and wound healing [5,6,8]. In India, various parts of the plant have been applied as traditional medicine for acidity, alopecia, asthma, gingivitis, edema, liver and spleen enlargement, urinary tract infection, snake bite, and scorpion sting, among others. In Pakistan, the leaves of the plant have been used for skin conditions and allergies [8].

A review of the literature on bioactive compounds derived from a medicinal plant can provide a deep understanding of the plant’s chemical composition, extraction optimization, selective isolation and identification techniques of the bioactive compounds, their pharmacological activities, and traditional knowledge validation. In this review, both the conventional and modern extraction, separation, isolation, identification, and characterization techniques of bioactive compounds derived from various parts of *E. alba* were discussed, and the pharmacological activities of the compounds for skin conditions (e.g., antibacterial, antifungal, antioxidant, and anti-inflammatory properties, as well as in vitro and in vivo cytotoxicity) were evaluated. Our review provides scientific insights into and a technological perspective on the efficient recovery of bioactive ingredients derived from *E. alba*, encouraging novel research directions and paving the way for the realization of innovative applications.

## 2. Materials and Methods

Relevant studies published before August 2023 were identified and selected from electronic databases, including Scopus, SciFinder, ScienceDirect, Google Scholar, and Wiley Library, using the following keywords: *Eclipta alba*, *Eclipta prostrata*, phytochemicals, extraction, separation, isolation, identification, characterization, pharmacological activity, and skin conditions. The retrieval was conducted from 23 June 2023 to 30 August 2023. Studies were selected if they met the following inclusion criteria: (i) original articles related to the extraction, separation, isolation, and identification methods, as well as the pharmacological activities of the main active ingredients for skin conditions; (ii) original articles that reported uses of commercial bioactive compounds of *E. alba* purchased from companies. The articles that used isolated pure compounds derived from other species, those that did not explore pharmacological activities related to skin conditions, and those that were not written in the English language were excluded. After evaluating the studies based on inclusion and exclusion criteria, 56 original research articles were discussed in this review.

## 3. Phytochemicals of *E. alba*

*E. alba* (L.) contains abundant bioactive substances, including phenolic compounds, alkaloids, triterpenes, flavonoids, coumestans, cardiac glycosides, saponins, and steroids. Among these classes of compounds, coumestans, flavonoids, thiopenes, and triterpenes were found to be prominent [10,11,12]. The bioactive compounds included in *E. alba* may differ depending on the environment, location of the sources, time of harvest, and period of storage [13]. In addition, secondary metabolism, responsible for the synthesis of therapeutic phytochemicals in medicinal plants, is influenced by environmental and cultivation conditions. Consequently, these factors collectively contribute to the distinctive chemical profiles observed in *E. alba* [14,15,16]. A summary of the bioactive substances found in various parts of *E. alba* is presented in Table 1.

### 3.1. Coumestans

Coumestans are naturally occurring isoflavonoids that are distinguished by their polycyclic aromatic backbone. Typically, they have a characteristic of the presence of oxygen in the heterocyclic four-membered ring containing a coumarin and benzofuran moiety linked by a double carbon bond. Naturally occurring coumestans possess multiple pharmacological functions against inflammation, cancer, diabetes, obesity, and aging and have demonstrated antimicrobial, antioxidant, UV-protective, and neuroprotective properties [25]. For example, wedelolactone and dimethyl wedelolactone were shown to be primarily coumestans found in *E. alba*.

### 3.2. Flavonoids

Flavonoids are phenolic compounds derived from benzo-γ-pyrone [26]. Flavonoids are well-known for their broad spectrum of pharmacological effects and are widely applied in nutraceutical, cosmetic, and pharmaceutical applications. Flavonoids can be categorized into flavonols, flavanonols, flavanols, flavones, flavanones, chalcones, and anthocyanins [27]. Several important bioactive flavonoids extracted from *E. alba* have been demonstrated, such as luteolin and luteolin-7-*O*-glucoside.

### 3.3. Thiopenes

Thiopenes (C_4_H_4_S) are heterocyclic compounds comprising a five-membered ring bearing one sulfur as a heteroatom [28]. These sulfur-containing molecules are rare in nature. Various genera are known as sources of thiopenes, including Asteraceae members *(Echinops, Eclipta, Pluchea, Artemisia, Ferula, Tagetes, Porophyllum, Atractylodes,* and *Xanthium*), fungi (*Penicillium*), and actinomycetes (*Streptomyces*). Among them, *Eclipta* was considered to be a rich source of thiopenes after *Echinops* [29].

### 3.4. Triterpenes

Triterpenes are complex cyclic compounds with six isoprene units as the skeleton, whereas a saponin is a glycoside with nonsugar aglycon, which can be classified as steroidal or triterpenes group [30]. Triterpenes (e.g., betulinic acid, betulin, and lupeol) have been shown to accelerate wound healing. A *Betulae* cortex extract containing betulin was tested in phase II clinical trials for surgical wound treatment and found to accelerate reepithelialization at skin graft sites [31]. Triterpenes and saponins are considered major compounds found in *E. alba*. Ursolic acid and eclalbasaponin exhibit interesting properties for the management of skin-related conditions and diseases.

### 3.5. Other Compounds

Various volatile components such as sitosterol (22.48%), *n*-undecane (21.34%), palmitic acid, methyl ester (14.45%), 2,4-diterbutylphenol (8.53%), and methyl stearate (7.81%) were also detected in *E. alba* [32]. Moreover, the presence of various phenolic compounds such as gallic acid, protocatechuic acid, chlorogenic acid [22], and steroidal alkaloids such as ecliptalbine and verazine [23] were reported in the *E. alba* leaf extract.

## 4. Pharmacological Activities of Phytochemicals Found in *E. alba* for Skin Care and Therapeutics

**Figure 2:**
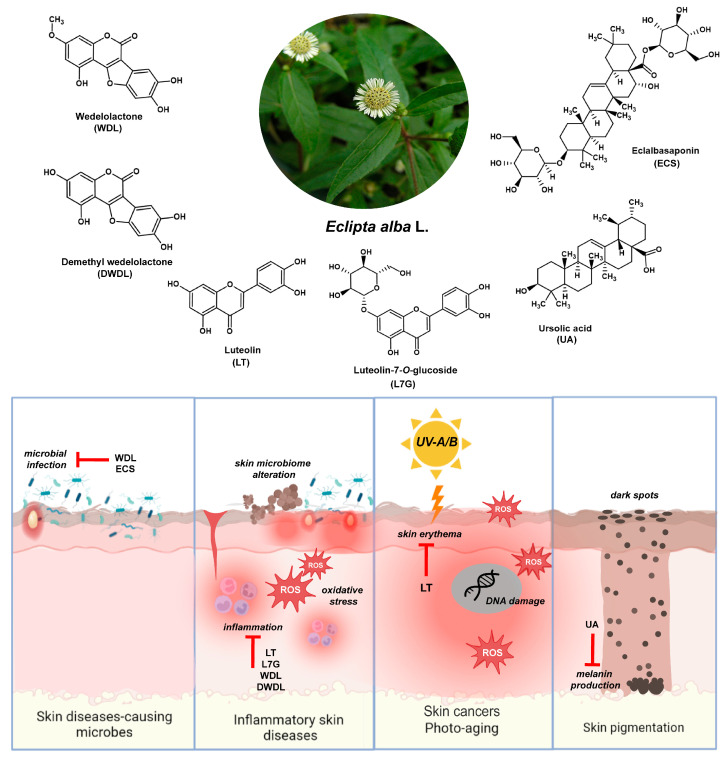
Bioactive phytochemicals found in *E. alba* possess pharmacological activities relevant to various skin conditions, such as skin disease-causing microbes, inflammatory skin diseases, skin cancers, photo-aging, and skin pigmentation. Major phytochemicals have been reported as anti-inflammatory agents, such as luteolin (LT), luteolin-7-*O*-glucoside (L7G), wedelolactone (WDL), and demethyl wedelolactone (DWDL). Isolated compounds, WDL and eclalbasaponin (ECS), are promising antimicrobial agents. The luteolin (LT) displayed bioactivities to suppress skin inflammation and erythema. Furthermore, ursolic acid (UA) demonstrated a promising action for encounter skin pigmentation by suppressing melanin production.

Phytochemicals derived from medicinal plants offer remarkable pharmacological activities against skin disease-causing microbes and inflammatory skin diseases. Skin infections can be caused by bacteria (e.g., acne, impetigo, scarlet, leprosy, boils, and scarlet fever), fungi (e.g., candidiasis, athlete’s foot, and fungal nail infection), and viruses (condylomata acuminata and herpes simplex labialis) [33,34]. In contrast, inflammatory skin conditions such as atopic dermatitis and psoriasis are primarily caused by the dysregulation of the immune system and microbial alteration in the skin microbiome [34]. Hence, medicinal plants used for certain skin diseases causing both microbial infection and inflammation perform various pharmacological activities, including wound healing, antibacterial, antifungal, antiviral, anti-inflammatory, antiproliferative, and photoprotective properties [33,34]. Here, we explored the antimicrobial, antioxidant, anti-inflammatory, and antimelanogenic properties of phytochemicals found in *E. alba* related to the management of various skin conditions and diseases (Figure 2).

### 4.1. Coumestans

#### 4.1.1. Wedelolactone

Wedelolactone was the first coumestan compound discovered and isolated from *Wedelia calendulacea* [25], and it is known as one of the major compounds derived from *E. alba* (0.36% in ethanolic extracts derived from a whole *E. alba* plant) [35]. Wedelolactone has been stated to exert many biological activities favorable for the treatment of skin conditions, such as antibacterial, antifungal, trypsin-inhibitory, antioxidant, and anti-inflammatory activities. For antibacterial activity, wedelolactone demonstrated its inhibitory action against American Type Culture Collection (ATCC) *Bacillus subtilis* and *Escherichia coli* at minimum inhibitory concentration (MIC) values of 500 and 1000 µg/mL (1591.14 and 3182.28 µM), respectively, whereas gentamicin showed MIC values of 2.5 and 5.0 µg/mL (5.23 and 10.46 µM, respectively) for both species [36]. It also inhibited the ATCC strain bacterial strains, such as *Staphylococcus aureus*, *Staphylococcus epidermidis*, and *Pseudomonas aeruginosa*, with MIC values of 250, 500, and 250 µg/mL, respectively. Further, it suppressed the growth of clinically isolated resistant bacteria, including ampicillin and penicillin-resistant *S. aureus*, with an MIC value of 125 µg/mL. Further, antifungal properties were shown against *Trichophyton rubrum* MYA-3108 and TruMDR2 strains with MIC values of >500 and 500 µg/mL, respectively (for comparison, fluconazole had an MIC value of 75 µg/mL for both fungal strains) [37].

Wedelolactone exhibited a promising property for the treatment of burns since it displayed trypsin-inhibitory activity with a half-maximal inhibitory concentration (IC_50_) value of 2.9 µg/mL compared to ovomucoid-derived hen egg white (IC_50_ = 1.45 µg/mL), which may suppress skin inflammation [38]. Further, it was found to help restore antioxidant enzymes in vitro. Wedelolactone suppressed nuclear factor kappa B (NFkB) induction and inflammation and altered the cell environment to prevent neoplastic transformation in mouse skin during ultraviolet B (UV-B) exposure [39]. In terms of cytotoxicity, wedelolactone was tested against HaCaT human keratinocytes with an IC_50_ value of 25.6 µg/mL and was found to be safer than a standard drug, paclitaxel (IC_50_ = 2.4 µg/mL). Furthermore, an ethanolic *E. alba* extract was shown to be less toxic to these cells, with an IC_50_ value of 271.4 µg/mL [22].

#### 4.1.2. Demethyl Wedelolactone

Demethyl wedelolactone was reported to be beneficial for burn treatment. Trypsin inhibitors are used to treat burns, and this protease is involved in inflammation and skin diseases. Demethyl wedelolactone inhibited trypsin at an IC_50_ value of 3 µg/mL [38].

### 4.2. Flavonoids

#### 4.2.1. Luteolin

Luteolin possessed antioxidant properties by scavenging the 2,2-diphenyl-1-picrylhydrazyl (DPPH) radical with an IC_50_ value of 12 µg/mL compared to *N*-acetylcysteine (IC_50_ = 32 µg/mL) and Trolox (IC_50_ = 25 µg/mL). Using the 2′,7′-dichlorodihydrofluorescein diacetate (H2DCFDA) assay for determining cellular reactive oxygen species (ROS) in UV-B-irradiated keratinocytes, luteolin was shown to be more effective than Trolox, possessing IC_50_ values of 3 µg/mL and 12 µg/mL, respectively. Furthermore, it inhibited UV-B-induced skin erythema as well as cyclooxygenase-2 (COX-2) and prostaglandin E_2_ (PGE_2_) expression in human skin by blocking the mitogen-activated protein kinase pathway, suggesting that luteolin has antioxidant, anti-inflammatory, and DNA-protective properties [40]. For lipopolysaccharide (LPS)-induced RAW 264.7 cells, luteolin inhibited nitric oxide (NO) production, PGE_2_, and COX-2 [41]. In comparison to paclitaxel (IC_50_ = 2.4 µg/mL), luteolin displayed cytotoxicity against HaCaT cells at 13.2 µg/mL [22].

#### 4.2.2. Luteolin-7-*O*-Glucoside

Luteolin-7-*O*-glucoside was demonstrated to be promising for the treatment of inflammatory skin diseases and hyperproliferative through the ability to neutralize the proliferation stimuli induced by interleukin (IL)-22 and IL-6 in HEKn human normal keratinocytes. In a mouse model of psoriasis, its topical administration decreased acanthosis and induced the expression of epidermal differentiation markers. It blocked the IL-22 signaling cascade and impaired the nuclear translocation of phosphorylated (activated) STAT3 [42]. In addition, it promoted skin epidermal stem cell proliferation by inducing beta-catenin and c-Myc expression and stimulated expanded potential stem cell migration, which has a role in skin injury repair. It was found that the epidermal thickness and alpha 6 integrin-positive and beta 1 integrin-positive cell numbers increased [43].

### 4.3. Thiopenes

#### Ecliprostin

*E. alba* was characterized by thiopenes as its constituents. Ecliprostin A–C, the thiopenes isolated from the aerial part of *E. alba*, exhibited antibacterial activity against *S. aureus* with MIC values of 25, 6.25, and 25 µM, respectively [11].

### 4.4. Triterpenes and Saponins

#### 4.4.1. Ursolic Acid

Ursolic acid is a pentacyclic triterpenoid carboxylic acid possessing pharmacological effects and low toxicity [44]. It inhibited skin pigmentation by promoting melanophagy in melanocytes. Ursolic acid suppressed the melanin content in Cellosaurus B16F1 cells treated with an α-melanocyte-stimulating hormone. Further, it increased melanosomal degradation [45]. Matrix metalloproteinase 2 (MMP-2) and oxidative were increased during UV-A irradiation. Ursolic acid was found to reduce the expression of MMP-2, ROS, and lipid peroxidation in UV-A-treated HaCaT cells. Moreover, it inhibited the expression of p53 gene. Ursolic acid was suggested to be beneficial for preventing aging stimulated by UV-A [46]. In UV-B-induced human lymphocyte cells, ursolic acid increased thiobarbituric-acid-reactive substances (TBARSs), lipid hydroperoxides, and the percentage of the DNA tail in UV-B-treated human lymphocyte cells. It also demonstrated antioxidative activity in vitro antioxidant assays [47].

#### 4.4.2. Eclalbasaponin

The antibacterial properties of eclalbasaponin were observed against *P. aeruginosa* and *B. subtilis*, with minimum bactericidal concentration values of 375 µg/mL and 187.5 µg/mL, respectively. Using Fourier-transform infrared spectroscopy (FTIR), scanning electron microscopy (SEM), and sodium dodecyl sulfate–polyacrylamide gel electrophoresis (SDS-PAGE) to observe the release of intracellular proteins, it was demonstrated to damage the bacterial cell membrane and cause cell death [48].

## 5. Extraction Methods

### 5.1. Conventional Methods for Extracting Bioactive Compounds from E. alba

Conventional extraction methods are widely used for the extraction of bioactive compounds from plants owing to their effectiveness, ease of implementation, cost-effectiveness, scalability, compatibility with different compounds, conservative approach, and long-standing knowledge base [49,50]. However, these conventional extraction techniques have limitations, such as lower extraction efficiency, longer extraction times, and potential environmental concerns [49,50]. In this section, the conventional extraction methods that have been applied to the extraction of bioactive compounds from *E. alba* are discussed.

#### 5.1.1. Solid–Liquid Extraction: Maceration, Agitation, and Percolation

The basic concepts and techniques used in maceration, agitation, and percolation for the extraction of crude pharmaceuticals include the leaching of soluble elements from solid materials with or without the aid of a mechanical factor or heat [51,52]. Diverse parts of the plant have been extracted using these extraction methods under various operating conditions, as summarized in Table 2. Petroleum ether–water, methanol, ethanol, water, and their mixtures have been used for the solid–liquid extraction of bioactive compounds from *E. alba* [22,53]. The methanolic solid–liquid extraction studies of bioactive compounds from the leaves of *E. alba* reported that the yields of wedelolactone were 5.1 mg/g at a solid-to-liquid ratio of 1:40 at 50 °C, agitated at 600 rpm for 15 h [54], and 0.41 mg/g at a solid-to-liquid ratio of 1:80 at 70 °C, agitated at 400 rpm for 90 min [55]. These two studies pointed out that the yield of bioactive compounds can be varied according to different operating conditions using the same solvent and part of the plant. Furthermore, Nakbanpote and co-workers reported the influences of drying methods and the effects of maceration and percolation using ethanol on phenolic compounds and antioxidant activity from *E. alba* leaves. It was found that phenolic contents and antioxidant activity were irrelevant to extraction techniques, and the freeze-drying method allowed the highest recovery of total phenolic content (TPC) and total flavonoid content (TFC), while thermal drying promoted degradation [22]. Notably, previous studies reported that wedelolactone was a major component found in methanol and ethanol extracts of the plant [22,56,57]. However, a recent study of the aqueous extraction of *E. alba* aerial parts of the plant reported that chlorogenic acid was the main active compound with no detection of wedelolactone in the extract [58]. Thus, previous findings suggest future research to elucidate the specific factors influencing the extraction process for better understanding of bioactive compounds profile of *E. alba* plant.

#### 5.1.2. Solid–Liquid Continuous Extraction: Soxhlet Extraction

Soxhlet extraction operates based on the principle of continuous solvent reflux, allowing for efficient target compound extraction [68,69]. This method has been used in the bioactive compound extraction process from *E. alba*, as described in Table 2. Several studies investigated the Soxhlet extraction of bioactive compounds from *E. alba* using different solvents (methanol, ethanol, water, and their mixtures, as well as hexane) [53,64,65], revealing varying yields of wedelolactone. In a previous study, the methanolic Soxhlet extract yielded the highest wedelolactone among different solvents (hexane, methanol, ethanol, and water) [65]. Furthermore, previous studies reported that Soxhlet extraction could provide the highest yield of wedelolactone compared to ultrasound-assisted extraction, heat reflux extraction, and other extraction methods [53,62]. In addition, previous studies of methanolic Soxhlet extraction of bioactive compounds from the leaves of *E. alba* reported that the yields of wedelolactone were 5.05 mg/g using a solid-to-liquid ratio of 1:150 at 90 °C for 6 h [54] and 0.7 mg/g using a solid-to-liquid ratio of 1:100 at 90 °C for 6 h [55]. These two studies used the same extraction parameters, including part of the plant, solvent, extraction temperature, and duration, but they differed in solid–liquid ratio. This variability suggests unidentified factors influencing the extraction process. Thus, elucidating the potential influencing factors is needed for a detailed mechanistic understanding of Soxhlet extraction on *E. alba* bioactive compounds that would contribute to optimizing extraction efficiency and reproducibility.

#### 5.1.3. Solid–Liquid Continuous Extraction: Reflux Extraction

Reflux extraction involves heating a solid sample with a heated solvent, and the extracted compounds are condensed and returned to the extraction vessel. Reflux extraction differs from Soxhlet extraction in that it does not include the Soxhlet extractor [70]. This method has been applied to bioactive compound extraction from various parts of *E. alba*, as described in Table 2. The ethanol–water and methanol–water systems have been used for the reflux extraction of bioactive compounds from *E. alba* [60,61]. In a comparative study of the extraction of luteolin from the aerial parts of *E. alba* using 80% ethanol at 80 ± 2 °C, acid reflux extraction yielded a higher amount of luteolin in a shorter extraction time compared to heat reflux extraction [61]. The yield of wedelolactone from aerial parts of the plant using an ethanol–water mixture through heat reflux extraction was reported to be 62.93 ± 0.82%, and the extraction duration was 5 h [60]. In addition, Fang and co-workers investigated the effect of heat flux extraction on wedelolactone yield from *E. alba* aerial parts using an ethanol–water system for 90 min, and the result showed that wedelolactone yield was 3.89 ± 0.11 mg/g [62]. In contrast, Zhao and co-workers explored the effect of heat flux extraction of wedelolactone yield from aerial parts of *E. alba* plant using a methanol–water system for 60 min, and the result showed that wedelolactone yield was 2.8 mg/g [63]. However, previous studies did not extensively explore optimization strategies for reflux extraction of bioactive compounds from *E. alba*, such as the use of response surface methodology or factorial designs, which could enhance efficiency.

#### 5.1.4. Liquid–Liquid Extraction: Aqueous Two-Phase Extraction (ATPE)

Aqueous two-phase extraction (ATPE) is a liquid–liquid extraction technique that involves using two immiscible aqueous phases, typically polymers or salts, instead of an organic solvent and water [71]. This method has been used in the extraction of bioactive compounds from the *E. alba* plant, as shown in Table 2. Gharat and Rathod applied ATPE for wedelolactone extraction from *E. alba* leaf. In this study, the impacts of molecular weight (MW) of polyethylene glycol (PEG) (4000–8000 MW), PEG concentration (12–18%), sodium citrate salt concentration (14–24%), and pH (5–8) on wedelolactone extraction were evaluated using central composite design (CCD) through response surface methodology (RSM). The yield of wedelolactone increased with an increase in the molecular weight of PEG. However, it reached over 6000 MW, and the yield decreased. PEG at a concentration of 18% was found to be able to create a sufficient hydrophobic reaction of the surface of the substance and PGE, leading to better partitioning. In addition, higher salt concentration was found to have a negative impact on ATPE for wedelolactone. At a neutral pH (pH7), it was reported to be the most suitable pH for the PEG/citrate salt system. The yield of wedelolactone through ATPE using optimal conditions of solid–liquid ratio of 1:40, PEG (6000 MW), PEG concentration (18% *w*/*v*), sodium citrate salt concentration (17.96% *w*/*v*), and pH 7 for 2 h was reported to be 6.52 mg/g, which was higher and needed a shorter time compared to that of Soxhlet extraction and batch extraction [54]. As there is a limited study of this method for *E. alba* plant, further investigation is needed to examine the scalability and reproducibility of the optimized conditions for the extraction of bioactive compounds from the *E. alba* plant.

#### 5.1.5. Hydrodistillation (HD)

Hydrodistillation (HD) is a method for extracting bioactive compounds, particularly essential oils, from plant materials [72]. A study conducted by Lin et al. explored the volatile compounds from the aerial parts of the *E. alba* plant using HD for 3 h. This study reported that 55 volatile compounds, including heptadecane, *n*-hexadecanoic acid, and pentadecane, were extracted from the aerial parts of the *E. alba* plant [66]. This study reported significant variations in the primary constituents in the essential oils of *E. alba* that had previously been documented. The principal constituents of *E. alba* leaves and stem bark were β-caryophyllene and α-humulene, whereas the stem bark had notable concentrations of (E)-β-farnesene. However, this study either included little or no of these chemicals. When compared to the aerial sections of *E. alba*, the primary constituents showed no appreciable variations. The variations in volatile components could result from genetic and environmental factors that influence the quality of medicinal plants [66].

### 5.2. Unconventional Extraction Methods for Extracting Bioactive Compounds from E. alba

Unconventional extraction methods have revolutionized bioactive compound extraction by offering higher extraction efficiencies, greater selectivity, reduced extraction time, and minimal solvent use [73,74,75]. As technology advances, these methods are expected to play a significant role in bioactive compound extraction and analysis. The unconventional extraction methods that have been used for the extraction of bioactive compounds from *E. alba* are described below.

#### 5.2.1. Ultrasound-Assisted Extraction (UAE)

Ultrasound-assisted extraction (UAE) uses cavitation and microstreaming to create alternating compression and rarefaction cycles in a solvent medium, causing mechanical disruption and cell wall rupture in plant material and facilitating the mass transfer of bioactive compounds [75,76,77]. This method has been used in the extraction of bioactive compounds from the *E. alba* plant, as shown in Table 2. Previous studies used methanol and ethanol–water mixtures for the UAE of bioactive compounds from *E. alba* [53,55,60]. For the recovery of wedelolactone from *E. alba*, Charpe and Rathod [55] and Fang and co-workers [62] carried out investigations to optimize the UAE process, assessing the influence of various parameters on wedelolactone yield. Charpe and Rathod reported that the highest yield of wedelolactone was obtained in the methanol extract, while Fang and co-workers reported that the highest wedelolactone yield was obtained in the ethanol–water extract. Extraction temperature had a positive significant impact on wedelolactone yield in both studies. Charpe and Rathod reported that extraction duration and extraction power had a positive significant impact on wedelolactone; in contrast, Fang and coworkers reported that these factors had a negative significant impact on wedelolactone yield. In addition, Fang and co-workers compared the extraction efficacy between UAE using a probe and using a sonication bath under the same operating parameters except for ultrasonic power; the result showed that UAE using a probe could provide a higher yield of wedelolactone, phenolic compounds, and antioxidant activity than UAE using sonication bath. In comparison with conventional methods, both studies reported that the yield of wedelolactone using UAE was comparable to or higher than that of conventional methods while requiring a shorter duration and minimal energy consumption. Conversely, Shi and co-workers compared the efficacy of ultrasound-assisted and microwave-assisted ethanolic extraction of wedelolactone from *E. alba* aerial parts using the same extraction parameters, and UAE demonstrated lower wedelolactone yield than that of microwave-assisted extraction [60]. Although previous studies proved that UAE could be a useful strategy in terms of time and energy for recovery of bioactive compounds from *E. alba* plant, the potential scalability of the laboratory-based UAE to industrial settings was not efficiently explored, representing a limitation in converting these promising results to larger-scale applications.

#### 5.2.2. Supercritical Fluid Extraction (SFE)

Supercritical fluid extraction (SFE) involves subjecting the solvent to high pressure and temperature, surpassing its critical point. This enhanced mass transfer enables highly efficient extraction of bioactive compounds from plant materials [78]. This method has been applied to the extraction of bioactive compounds from various parts of the *E. alba* plant, as described in Table 2. Liquid carbon dioxide (CO_2_) has been used as an extracting solvent [53,64]. A previous study of the supercritical carbon dioxide extraction of *E. alba* reported that it produced a higher yield of wedelolactone (15.37 ± 0.63 mg/100 g) and took a shorter extraction time (60 min) than conventional Soxhlet extraction (13.71 ± 0.82 mg/100 g and 24 h, respectively) [64]. In contrast, in a study conducted by Savita et al., supercritical carbon dioxide extraction of the whole *E. alba* plant was optimized using different pressures (4000–6000 psi) and temperatures (40–50 °C), and the highest wedelolactone yield was obtained at a pressure of 6000 psi and temperature of 5 °C. However, the resultant wedelolactone yield from SFE was the lowest among six different extraction methods [53]. Future studies need to examine how pressure and temperature variations affect the extraction efficiency and yield of specific bioactive compounds from *E. alba*.

#### 5.2.3. Microwave-Assisted Extraction (MAE)

Microwave-assisted extraction (MAE) uses microwave irradiation to enhance mass transfer and extraction efficiency by utilizing dielectric heating, thermal gradients, and cell wall disruption [75,79]. This technique has been applied to the extraction process of bioactive compounds from various parts of the *E. alba* plant, as described in Table 2. Previous studies used methanol and ethanol–water mixtures for the MAE of bioactive compounds from *E. alba* [53,60]. Shi and co-workers explored the effect of microwave-assisted ethanolic extraction of wedelolactone from aerial parts of the plant and optimized extraction conditions (the irradiation power of 100–300 W, solid–liquid ratio of 1:20–1:40, ethanol concentration of 60–100%, extraction time of 15–35 min) through CCD in RSM. This study reported that all four variables had a positively significant effect on wedelolactone yield. Furthermore, this study reported that the interaction between irradiation power and ethanol concentration and the interaction between solid–liquid ratio and extraction duration had a negatively significant impact on wedelolactone yield. In contrast, the interaction between irradiation power and the other two variables (solid–liquid ratio and extraction time) had a positively significant impact on wedelolactone yield. Under the optimal extraction conditions of irradiation power of 208 W, ethanol concentration of 90%, solid–liquid ratio of 1:33, and extraction duration of 26.5 min, the resultant wedelolactone yield was 82.67 ± 0.16%. In comparison with ultrasonic and conventional extraction methods (heat reflux extraction and maceration), MAE demonstrated the highest wedelolactone yield and took less time compared to these methods [60]. Conversely, Savita and co-workers conducted microwave-assisted methanolic extraction of wedelolactone from *E. alba* whole plant, and the resultant wedelolactone yield (0.27%) was higher than supercritical carbon dioxide extraction (0.013%); however, it was lower than UAE (0.36%), Soxhlet extraction (0.48%), and other conventional methods [53]. As previous studies used methanol and ethanol as solvents for microwave-assisted extraction, it suggests future research to explore MAE with alternative solvents (e.g., polyols, deep eutectic solvents), which have less environmental impact and safety concerns.

#### 5.2.4. Ultrahigh Pressure-Assisted Extraction (UHPE)

Ultrahigh pressure-assisted extraction (UHPE) is a modern technique for isolating bioactive compounds from plant materials [63]. The method has been applied to the extraction of bioactive compounds from various parts of the *E. alba* plant, as described in Table 2. Zhao and co-workers proved that UHPE, in combination with high-speed counter-current chromatography, was an efficient combination for the extraction and purification of bioactive compounds from the *E. alba* plant. In this study, the effects of methanol–water mixture (80–100%), liquid–solid ratio (10:1–30:1 mL/g), extraction pressure (100–300 MPa), and duration (3–9 min) on the yields of bioactive compounds were explored through an orthogonal design. This study reported that the solvent concentration had the highest significant impact; meanwhile, the extraction duration had a negligible effect on the yield of bioactive compounds. Under optimal extraction, parameters of 80% methanol, liquid–solid ratio of 20:1, extraction pressure of 100 MPa, and extraction duration of 3 min, the yields of wedelolactone and isodemethylwedelolactone were reported to be 2.4 and 0.7 mg/g, respectively, which were comparable to their yields obtained from the heat reflux extraction (2.8 and 0.8 mg/g, respectively) [63]. However, further investigation is needed to examine the wider applicability of UHPE across varying plant sources and conditions.

### 5.3. Combinatorial Processes for Extracting Bioactive Compounds from E. alba

A combination of extraction processes offers a valuable approach for minimizing the operation expenses and adverse effects on human health and the environment and obtaining comprehensive and high-quality bioactive compound extracts [74]. However, few studies have been performed using the combination of two or more extraction methods to extract bioactive components derived from *E. alba*.

#### 5.3.1. Maceration-Percolation

A previous study of the extraction of wedelolactone from a whole plant of *E. alba* using six different extraction methods showed that maceration (24 h), followed by percolation (until percolates became colorless), demonstrated a higher yield of wedelolactone (0.38%) than MAE, ultrasonication extraction, orbital shaker bath extraction, and SFE and a lower wedelolactone yield than that of Soxhlet extraction (0.48%). However, regarding extraction time, it took the longest duration among the six different extraction methods [53].

#### 5.3.2. Ultrasound-Assisted Microwave Extraction (UAME)

A study conducted by Yi et al. reported that ultrasound-assisted microwave extraction (UAME) is an efficient method with higher yield and shorter duration for the extraction of luteolin from the aerial parts of the *E. alba* plant. In this study, extraction solvents were screened, and 80% ethanol was found to yield the highest luteolin among ethanol, methanol, 80% methanol, and water. Interestingly, this study revealed that the addition of hydrochloric acid to aqueous ethanol up to a certain ratio could enhance the yield of luteolin. Afterward, the extraction conditions, solvent concentration (20–80%), solid–liquid ratio (1:20–1:50), extraction power (10–40 W), and extraction duration (3–6 min), were optimized through the orthogonal test, and the solvent concentration was found to be the most influent factor among independent variables. Under optimal extraction conditions of 80% ethanol with the presence of hydrochloric acid, solid-to-liquid ratio 1:50 (*w*/*v*), microwave power of 40 W, and extraction duration of 3 min for three cycles, UAME demonstrated a higher yield of luteolin in a shorter period (0.690 mg/g, 9 min) than heat reflux extraction (0.676 mg/g, 240 min) and reflux acid extraction (0.689 mg/g, 60 min) [61].

#### 5.3.3. Combination of Other Extraction Methods

At present, the use of a combination of other extraction methods for *E. alba* is limited. However, several investigations on the combined use of extraction techniques from various plants have been reported, including enzyme-based ultrasound-microwave-assisted extraction (EUMAE), SFE-pressurized liquid extraction (PLE), SFE-UAE, supercritical fluid extraction assisted with enzyme (SFE-EE), and PLE assisted with enzyme (PLE-EE) [74,80,81]. These methods are recommended for future research on the extraction of bioactive compounds from the *E. alba* plant.

## 6. Separation and Purification Technologies

The separation and purification of bioactive components are crucial steps in both medicinal research and the discovery of novel natural products. Further details on these procedures for isolating and purifying phytochemicals from *E. alba* are described below.

### 6.1. Column Chromatography (CC)

Column chromatography (CC) is a simple, inexpensive, and effective method and is widely used for separating natural bioactive compounds. Previous studies that have conducted CC are summarized in Table 3. In previous studies, wedelolactone was isolated by silica gel CC eluted with toluene [53], methanol–chloroform [65], and by Sephadex LH-20 CC eluted with methanol [10]. In another study reported by Liu et al., after fractionation of the extract using a series of CC including microporous adsorption resin and silica gel coupled with thin layer chromatography (TLC), eclalbasaponin I, and luteolin, were isolated using Sephadex LH-20 eluted with chloroform–methanol and luteolin-7-*O*-glucoside was isolated using octadecylsilyl column eluted with methanol–water [10]. Similarly, after a series of columns was used for fractionation of the extract, a silica gel column eluted with dichloromethane–methanol mixture was used for isolation of three new thiophene derivatives, ecliprostins A–C, [11], and octadecyl-silica (ODS); Sephadex LH-20 columns eluted with methanol/methanol–water mixture were used for isolation of three new olean-type triterpenoid saponins [12] and eight bioactive compounds, including eclalbasaponin I and IV from the aerial parts of *E. alba* [82] in combination with semipreparative/preparative high-performance liquid chromatography. Previous studies did not explain the rationale behind selecting specific solvent systems, suggesting further exploration of solvent optimization could improve the reproducibility and efficiency of the isolation process.

### 6.2. High-Performance Liquid Chromatography (HPLC)

High-performance liquid chromatography (HPLC) is one of the most effective instruments in analytical chemistry at present and is frequently used to analyze medicinal and pharmaceutical products both quantitatively and qualitatively [85]. Previous studies performed using HPLC are summarized in Table 3. In previous studies, reverse phase C18 HPLC was mainly used to isolate bioactive compounds from the *E. alba* plant [10,55,61,62]. The mobile phases for reverse phase C18 HPLC included methanol–water with or without the addition of acetic acid, acetonitrile–water containing formic acid, and 0.5% aqueous glacial acetic acid for separation of wedelolactone [60,62,64,83], and methanol containing phosphoric acid and acetonitrile containing formic acid for separation of luteolin from the *E. alba* plant [61,84]. The study conducted by Chan et al. reported that chlorogenic acid was detected as one of the major compounds using the HPLC analysis of the *E. prostrata* aqueous extract [58]. In addition, three new thiophene derivatives, ecliprostins A–C [11]; three new olean-type triterpenoid saponins [12]; and eight bioactive compounds, including eclalbasaponin I and IV [82] isolated from the aerial parts of *E. alba* using a series of column chromatography, were further purified using semipreparative/preparative HPLC. In addition, previous studies evaluated and validated reversed-phase HPLC [83,86,87] as a quantitative measurement method for wedelolactone extracted from *E. alba*.

### 6.3. High-Speed Counter-Current Chromatography (HSCCC)

High-speed counter-current chromatography (HSCCC) is a novel liquid–liquid partition chromatography technique that uses two liquid phases for separating bioactive chemicals. Since solid support is not used in HSCCC, sample loss through irreversible adsorption and degradation at the solid–liquid interface is avoided [88]. It has been widely used in the separation and analysis of bioactive compounds in combination with column chromatography [89]. Zhao and co-workers conducted UHPE in combination with HSCCC for the extraction and isolation of bioactive compounds from the *E. alba* plant. This study reported that solvent systems play a crucial role in the separation of bioactive compounds using HSCCC. Based on the chemical characteristics of desired compounds, the solvent systems, including petroleum ether, ethyl acetate, methanol, and water, in different volumetric ratios, were chosen and optimized. This study reported that HSCCC using a solvent system of petroleum ether–, ethyl acetate–, and methanol–water in the ratio of 3:7:5:5 (*v*/*v*) demonstrated three chromatographic peaks and subsequent HPLC analysis revealed that bioactive compounds, including wedelolactone, isodemethylwedelolactone, and luteolin with high purities (over 95%) were isolated in a single-step separation [63].

### 6.4. Other Methods for Separation and Purification of Bioactive Compounds

The use of other separation and purification techniques of bioactive compounds from *E. alba* is still limited. However, other techniques were used to separate and purify bioactive substances from plants, including affinity chromatography, electrophoresis, supercritical fluid chromatography (SFC), and nano-liquid chromatography (nano-LC) [90,91,92,93]. These methods are recommended for the separation and purification of bioactive compounds in *E. alba*.

## 7. Characterization Techniques

Exploring the medical potential of a plant extract, finding new drugs, developing formulations, and ensuring product quality all depend on the identification and characterization of bioactive components in that extract. This scientific information not only broadens our understanding of conventional herbal remedies but also opens the path for the innovation of more potent therapeutics. The techniques employed to find bioactive substances in *E. alba* are listed in Table 4 and briefly reviewed below.

### 7.1. Ultraviolet–Visible Spectroscopy (UV–Vis)

Ultraviolet–visible spectroscopy (UV–Vis) is a widely used technique for identifying and characterizing bioactive compounds in plant extracts. It relies on the absorption of UV light by compounds with conjugated double bonds or aromatic ring systems in the UV and visible regions of the electromagnetic spectrum, typically in the range of 200–700 nm [101]. Previous studies that have used UV–Vis spectroscopy are summarized in Table 4. The structure of a compound can be inferred from the UV–Vis spectrum, but its value increases when supplemented with information from infrared (IR) and nuclear magnetic resonance (NMR) spectra [102]. Previous studies conducted UV–Vis spectroscopy in combination with IR spectroscopy and NMR spectroscopy for the identification and characterization of bioactive compounds from *E. alba* plant extract [12,82,84]. UV–Vis spectroscopy detected the absorption maxima (*λ*max) of the isolated luteolin using methanol at 268 and 345 nm [84]. The UV spectra of three new olean-type triterpenoid saponins isolated from the 80% ethanol extract of the aerial parts of the *E. alba* plant were recorded [12]. Furthermore, Han and co-workers used UV–Vis spectroscopy to detect the UV spectra of eight bioactive compounds, including eclalbasaponin I and IV, isolated by column chromatography from the extract of aerial parts of *E. alba* plant [82].

### 7.2. Thin Layer Chromatography (TLC) and High-Performance Thin Layer Chromatography (HPTLC)

Thin layer chromatography (TLC) is a widely applied chromatographic method for the separation and identification of bioactive substances by comparing the retention factor (Rf) of these bioactive substances [103]. Previous studies conducted using TLC and HPTLC are summarized in Table 4. Tambe and co-workers were able to characterize luteolin isolated from methanolic extract of the *E. alba* leaves using TLC plates, methanol–ethyl acetate–formic acid–toluene as an effluent, and polyethylene glycol (PEG) as a spraying agent [84]. In addition, a previous study used TLC to identify the presence of wedelolactone in the ethyl acetate fraction of methanolic extract of whole *E. alba* plant [94]. HPTLC provides greater performance and improved resolution compared to TLC [104]. A recent study used HPTLC for the analysis of bioactive compounds using TLC plates, ethyl acetate–formic acid–toluene as effluent, and two reagents (ethanolamine diphenyl borate and polyethylene glycol) as spraying agents, and the findings of HPTLC indicated the presence of wedelolactone, luteolin, chlorophyll, and other non-phenolic compounds [22]. Furthermore, Savita and co-workers attempted to quantify wedelolactone content from various extracts of the plant using HPTLC. A pre-activated silica gel HPTLC plate (60 F 254, 20 × 10 cm) was used as a stationary phase, and a mixture of toluene–ethyl acetate (9:1 *v*/*v*) was used as a mobile phase. This study conducted the method validation by evaluating the limit of detection, the limit of quantitation, the range of linearity, precision, and accuracy. The validation results of this HPTLC method attract great industrial applicability as a precise, rapid, and selective method for estimation of wedelolactone [53]. Previous studies of TLC revealed the detection of wedelolactone, luteolin, and chlorophyll in *E. alba*, but there is limited exploration of other bioactive compounds such as saponins and triterpenes using TLC.

### 7.3. Fourier Transform Infrared Spectroscopy (FT–IR)

Infrared spectroscopy (IR) uses infrared radiation to provide information about functional groups and chemical bonds, aiding in the identification and characterization of bioactive compounds. Fourier transform infrared spectroscopy (FT–IR) is a special of IR spectroscopy in which a detector measures the light, which is converted into an interferogram using Fourier transform [105,106,107]. Previous studies that have used IR spectroscopy are summarized in Table 4. Muruganantham and co-workers conducted a comparative FT–IR analysis between the stem, leaf, and root parts of *E. alba* Hassk. and *E. prostrata* Linn using BRUKER IFS 66 model FT–IR spectrometer with standard KBr technique. This study reported that carboxylic acid is the major functional group in both plants. In addition, both plants are rich in amino derivative groups, sulfur groups, and other functional groups, including polysaccharides and nitrates, which are accountable for the medicinal values of both plants [24]. Furthermore, a recent study of the aerial parts of *E. prostrata* used FT–IR spectroscopy accompanied by NMR and liquid chromatography–mass spectrometry (LC–MS) to identify the chemical constituents of the plant extract [82]. In this study, a new compound, 7-*O*-methylorobol-4′-*O*-β-D-glucopyranoside, was discovered, and its functional groups detected in an IR spectrum included hydroxy group, aromatic ring, α, β-unsaturated ketone, and an *O*-glycosidic linkage.

### 7.4. Scanning Electron Microscopy with Energy-Dispersive X-Ray Spectroscopy (SEM–EDS)

Scanning electron microscopy with energy-dispersive X-ray spectroscopy (SEM–EDS) involves SEM with an electron beam, resulting in high-resolution images of the sample topography based on backscattered electrons. EDS, alternatively, detects and measures characteristic X-rays emitted by the sample, allowing for element identification by analyzing the energies of these X-rays [108,109]. Previous studies that have used SEM–EDS are summarized in Table 4. Muruganantham and co-workers conducted a comparative SEM–EDS analysis between the stem, leaf, and root parts of *E. alba* Hassk. and *E. prostrata* Linn using SEM instrument at 20 kV and high vacuum mode. The study reported that *E. alba* contained a larger content of beneficial elements like sodium, magnesium, potassium, calcium, copper, zinc, and iron but less harmful components such as cadmium than *E. prostrata*. Therefore, this study concluded that *E. alba* Hassk. is a more effective and safe herbal plant compared to *E. prostrata* Linn [24].

### 7.5. Nuclear Magnetic Resonance Spectroscopy (NMR)

Nuclear magnetic resonance spectroscopy (NMR) is based on the interaction of radiofrequency (RF) radiation and a strong magnetic field with atomic nuclei, and they play a crucial role in the structural elucidation of isolated natural compounds and synthetic compounds [110,111]. Previous studies that have used NMR are summarized in Table 4. The combined use of ^1^H-NMR and ^13^C-NMR, which are complimentary methods, improves the comprehension of the structure and constitution of molecules in organic chemistry [112]. Liu et al. reported NMR spectra of wedelolactone, eclalbasaponin I, luteolin, and luteolin-7-*O*-glucoside using ^1^H-NMR and ^13^C-NMR [10]. In this study, ^1^H-NMR using deuterated dimethyl sulfoxide (DMSO-d_6_) as a solvent determined the chemical shift (δ) values of all hydrogens of aforementioned bioactive compounds; meanwhile, ^13^C-NMR, using deuterated dimethyl sulfoxide as a solvent, determined the δ values of all carbons of these bioactive compounds indicating the types of atoms present and their chemical environment. Moreover, a recent study used NMR using DMSO as one of the characterization techniques to identify luteolin [84]. In addition, Yu and co-workers identified and compared the structures of ecliprostins A, B, and C isolated from the aerial part of *E. alba* using 1D NMR, including ^1^H-NMR and ^13^C-NMR in deuterated chloroform as a solvent coupled with LC-MS; they confirmed the whole structure of the aforementioned compounds using 2D NMR, including ^1^H-^1^H- homonuclear correlation spectroscopy (COSY) and heteronuclear multiple-bond correlation spectroscopy (HMBC) [11]. This technique revealed the structural similarities and chemical shift variations between these three ecliprostins, and this study contributed a novel finding that ecliprostin C is a symmetrical dimer of ecliprostin A.

### 7.6. Liquid Chromatography Coupled with Mass Spectrometry (LC–MS)

Liquid chromatography coupled with mass spectrometry (LC–MS) offers high sensitivity, accurate mass determination, and structural elucidation of bioactive compounds [113]. Previous studies that have used LC–MS are summarized in Table 4. A recent study used quadrupole time-of-flight mass spectrometry (Q–TOF–MS) to identify luteolin [84]. Moreover, LC–MS coupled with NMR identified three new thiophene derivatives, ecliprostins A–C, isolated from the aerial parts of *E. alba* [11]. Han and co-workers evaluated LC-MS-based qualitative and quantitative analysis of bioactive compounds from aerial parts of the *E. alba* plant. This study optimized the chromatographic parameters of liquid chromatography–triple-quadrupole mass spectrometry (LC–QQQ–MS), including mobile phase and column temperature, and reported that acetonitrile–water (with 0.1% formic acid) and a 35 °C column temperature demonstrated good resolution of adjacent peaks. In addition, LC-quadrupole time-of-flight MS (LC–Q–TOF–MS) was used to identify nine bioactive compounds, including wedelolactone ([M − H]^−^ = 313.0363), ecliptasaponin C ([M + HCOO]^−^ = 841.4590), luteolin ([M − H]^−^ = 285.0409), eclalbasaponin IV ([M − H]^−^ = 795.4550), apigenin ([M − H]^−^ = 269.0451), ecliptasaponin A ([M − H]^−^ = 633.4023), and other compounds from the plant extract [96]. Furthermore, this study conducted the method validation for quantitative analysis by evaluating the limit of detection, the limit of quantitation, the range of linearity, precision, and accuracy. The validation results demonstrated that this LC–MS approach was sufficiently sensitive, exact, and accurate to allow for the simultaneous quantitative assessment of the nine compounds from the aerial portion of *E. alba*. Therefore, these two distinct LC–MS analytical techniques were proved to be able to accomplish rapid profiling and determination of the main components of *E. alba* [96].

### 7.7. Gas Chromatography Coupled with Mass Spectrometry (GC–MS)

Gas chromatography coupled with mass spectrometry (GC–MS) is commonly used to analyze volatile bioactive compounds, such as essential oils, monoterpenes, and fatty acids [114,115]. Previous studies that have used GC–MS are summarized in Table 4. Sahoo and co-workers studied GC–MS analysis of volatile substances in the essential oil obtained from the aerial part of *E. alba*. This study reported that 59 volatile compounds have been identified, and β-pinene, caryophyllene, and α-humulene were abundant in the essential oils obtained from the aerial part of *E. alba* [97]. Previous GC–MS studies of the methanolic leaf extract of *E. alba* using helium gas as a carrier gas identified eight bioactive compounds [100] and seven bioactive compounds [98], as listed in Table 4, and these two studies reported the same bioactive compounds including c-sitosterol, oleic acid, eicosyl ester, 10-octadecenoic acid, and methyl ester. Furthermore, Chuahan and co-workers studied GC–MS analysis of ethyl acetate, methanol, and water extracts of the aerial part of the plant and reported that the extracts contained various bioactive substances, including glycine, hydrazine carboxyamide, garbamic acid, naphthoquinone, and other substances [99].

## 8. Strengths and Limitations of the Current Review

**Figure 3:**
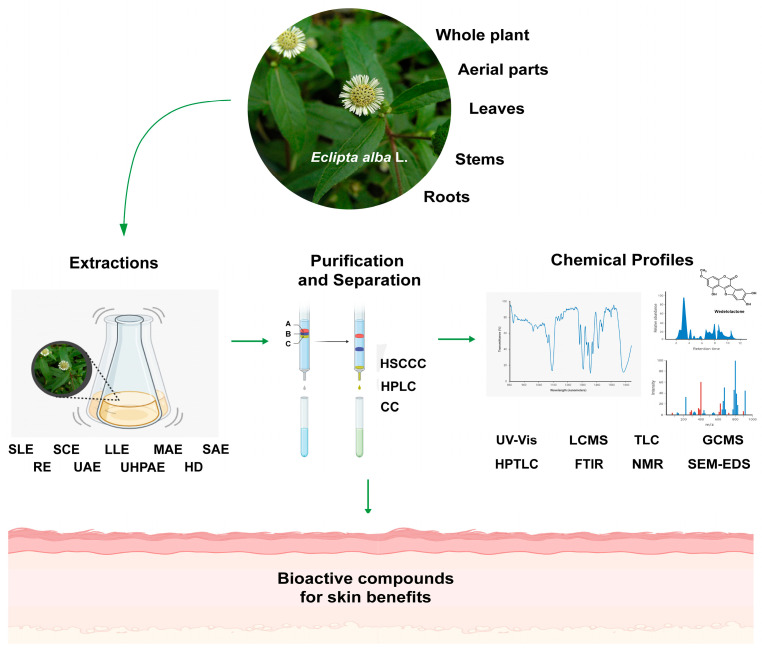
Summary of different technologies used for the extraction, purification, and identification of bioactive compounds in *E. alba*. Both conventional and modern extraction techniques have been reported to explore the bioactive phytochemicals for skin benefits (SLE: solid–liquid extraction; SCE: solid–liquid continuous extraction; LLE: liquid–liquid extraction; MAE: microwave-assisted extraction; SAE: Soxhlet-assisted extraction; RE: reflux extraction; UAE: ultrasound-assisted extraction; UHPAE: ultrahigh pressure-assisted extraction; and HD: hydrodistillation). Further, numerous purifications, separations, characterization, and phytochemicals profiling techniques were utilized to isolate and identify the bioactive compounds from *E. alba,* including high-speed counter-current chromatography (HSCCC), high-performance liquid chromatography (HPLC), column chromatography (CC), ultraviolet–visible spectroscopy (UV–Vis), liquid chromatography–mass spectrometry (LC–MS), gas chromatography–mass spectrometry (GC–MS), thin-layer chromatography (TLC), high-performance thin-layer chromatography (HPTLC), Fourier transform infrared spectroscopy (FT–IR), nuclear magnetic resonance spectroscopy (NMR), and scanning electron microscopy with energy-dispersive X-ray spectroscopy (SEM–EDS).

Up-to-date extraction, separation, and identification methods of bioactive compounds from *E. alba* and the antimicrobial, antioxidative, and anti-inflammatory activities of phytochemicals identified in *E. alba* linked to the therapy of numerous skin diseases and conditions were reviewed (Figure 3), and the limitations identified in the existing literature were highlighted. However, our review has a few limitations worth mentioning. Due to the use of electronic databases, it may exclude studies not indexed or published in peer-reviewed journals. The use of specific keywords in search results can potentially exclude relevant studies that use different terminology to describe similar concepts. Additionally, the focus on pharmacological activities related to skin conditions may exclude studies investigating broader effects not directly related to skin health, potentially limiting the understanding of the overall pharmacological potential of bioactive compounds derived from *E. alba*.

## 9. Conclusion and Perspectives

*E. alba* contains a wide variety of bioactive substances, primarily alkaloids, triterpenes, flavonoids, coumestans, cardiac glycosides, saponin, and steroids. These isolated compounds derived from *E. alba*, in particular, wedelolactone and derivatives, are well-characterized, and coumestans are the main bioactive compounds that possess various skin-related pharmacological activities. These bioactive compounds can be valuable resources in developing skin-related medications, for example, anti-aging, anti-septic, and wound healing formulations, from natural sources. To produce high-quality bioactive component extracts from *E. alba*, effective and cutting-edge extraction technologies and potential combinations of extraction methods are suggested to be investigated. Further studies are suggested to involve innovative techniques, such as nano-LC and SFC on the separation and purification, and hyphenated techniques on the identification of bioactive chemicals in *E. alba*. In-depth mechanistic investigations to comprehend the molecular processes behind the therapeutic benefits of *E. alba* bioactive substances on skin disorders ought to be the focus of future studies. Clinical trials and human studies should be incorporated to confirm the safety and effectiveness of *E. alba* bioactive substances in treating certain skin problems, guaranteeing realistic and evidence-based therapeutic applications. Overall, *E. alba* is a promising natural source of a variety of bioactive chemicals, and understanding their extraction, separation, and identification techniques will offer a technological perspective on the efficient recovery of bioactive chemicals from *E. alba*, paving the way for innovative research areas and its practical applications including the development of cosmeceutical and skincare products, anti-inflammatory agents, and formulations for dermatological treatments.

## Figures and Tables

**Table 1:** Phytochemicals found in various parts of *E. alba*.

Parts of Plant	Types	Compounds	References
Whole plants	Triterpenes	Eclalbasaponin, ursolic acid, α-amyrin, eclalbatin	[7]
	Flavonoids	Orobol, oroboside	[17,18]
	Thiopenes	5-Hydroxymethyl-terthienyl tiglate, hydroxymethyl-terthienyl acetate, ecliptal hydroxymethyl-terthienyl agelate	[17]
	Coumestans	Isodemethylwedelolactone, demethylwedelolactone Wedelolactone,	[17,18]
Aerial parts	Triterpenes	3-*O*-(2-*O*-Acetyl-β-D-glucopyranosyl) oleanolic acid-28-*O*-(β-d-glucopyranosyl) ester, 3-*O*-(β-D-glucopyranosyl) oleanolic acid-28-*O*-(6-*O*-acetyl-β-D-glucopyranosyl) ester, and 3-*O*-(6-*O*-acetyl-β-D-glucopyranosyl) oleanolic acid-28-*O*-(β-D-glucopyranosyl) ester (three new triterpenes), eclalbasaponin, echinocystic acid, 3-oxo-16α-hydroxy-olean-12-en-28-oic acid	[12,19,20]
	Saponin	Echinocystic acid-3-*O*-(6-*O*-acetyl)-β-D-glucopyranoside	[19]
	Flavonoids	Apigenin, luteolin, pratensein, pratensein-7-*O*-β-D-glucopyranoside, diosmetin, 3′-hydroxybiochanin A, 3′-*O*-methylorobol	[19,21]
	Thiopenes	5′-Isovaleryloxymethyl-5-(4-isovaleryloxybut-1-ynyl)-2,2′-bithiophene, 5-methoxymethyl-2,2′:5′,2″-terthiophene, 3′-hydroxy-2,2′:5′,2″-terthiophene3′-*O*-β-D-glucopyranoside	[19,20]
	Coumestans	Wedelolactone, demethyl wedelolactone	[19]
Leaves	Coumestans	Wedelolactone, dimethylwedelolactone sulfate	[22]
	Triterpenes	Eclalbasaponin	[22]
	Phenolic	Gallic acid, protocatechuic acid, chlorogenic acid, protocatechualdehyde	[22]
	Flavonoids	Luteolin, luteolin-7-*O*-glucoside, 3-hydroxybiochanin A	[22]
	Steroidal alkaloids	Ecliptalbine, verazine, hydroxyverazine, epi-verazine	[23]
Stems	Coumestans	Wedelolactone	[24]
Roots	Coumestans	Wedelolactone	[24]

**Table 2:** Summary of extraction techniques used in bioactive compound extraction from *E. alba*.

Extraction Technique	Part of the Plant	Extraction Solvent	Optimal Extraction Parameters	Phytochemical Content	Identification Procedure	Main Results	References
Solid–liquid extraction	Roots	Petroleum ether-water system	50 g of powder using hydroalcoholic solvent for 48 h	Phytochemicals	Phytochemical screening	Yield (2.5% *w*/*v*), phenols, saponin, diterpene	[59]
	Leaves	Ethanol	Freeze-dried sample, the solid–liquid ratio (SLR) of 1:100, shaking at 150 rpm for 24 h at room temperature	Total phenolics	LC–ESI–QTOF–MS/MS	TPC (20.3 ± 2.4 mg GA/g), wedelolactone, luteolin, other phenolic compounds	[22]
	Aerial parts	Ethanol–water system	SLR of 1:33 of 90% ethanol in water for 24 h	Wedelolactone	HPLC	Wedelolactone yield (67.79 ± 2.59%)	[60]
	Leaves	Methanol	SLR of 1:40 at 50 °C, 600 rpm for 15 h	Wedelolactone	HPLC	Wedelolactone yield (5.1 mg/g)	[54]
	Leaves	Methanol	SLR of 1:80 at 70 °C, 400 rpm for 90 min	Wedelolactone	HPLC	Wedelolactone yield (0.41 mg/g)	[55]
	Aerial parts	Water	SLR of 1:2 at 100 °C for 20 min	Total phenolics	HPLC	TPC (176.45 ± 11.56 mg GAE/g sample), CGA (1.75 ± 0.01 mg/g sample)	[58]
	Whole plants	Methanol	SLR of 1:5 for 3 h	Wedelolactone	HPTLC	Wedelolactone yield (0.48% *w*/*w*)	[53]
	Leaves	Ethanol	Freeze-dried sample, SLR of 1:100, the flow rate at 0.1–0.2 mL/min using disposal syringe	Total phenolics	LC–ESI–QTOF–MS/MS	TPC (20.3 ± 0.9 mg GA/g), wedelolactone, luteolin, other phenolic compounds	[22]
Reflux extraction	Aerial parts	Ethanol–water system	SLR of 1:150 of 80% ethanol in water, containing 1.8 mL hydrochloric acid at 80 ± 2 °C for 60 min	Luteolin	HPLC	Luteolin (0.689 mg/g)	[61]
	Aerial parts	Ethanol–water system	SLR of 1:40 of 70% ethanol in water for 90 min	Total phenolics, wedelolactone	HPLC	Wedelolactone yield (3.89 ± 0.11 mg/g), TPC (18.20 ± 0.51 mg/g)	[62]
	Aerial parts	Ethanol–water system	SLR of 1:75 of 80% ethanol in water at 80 ± 2 °C for 120 min	Luteolin	HPLC	Luteolin (0.676 mg/g)	[61]
	Aerial parts	Ethanol–water system	SLR of 1:33 of 90% ethanol in water for 5 h	Wedelolactone	HPLC	Wedelolactone yield (62.93 ± 0.82%)	[60]
	Aerial parts	Methanol–water system	SLR of 1:20 using 80% methanol in water at 85 °C for 60 min	Wedelolactone	HPLC	Wedelolactone yield (2.8 mg/g) isodemethylwedelolactone yield (0.8 mg/g)	[63]
Soxhlet assisted extraction	Whole plants	Methanol	SLR of 1:30 for 24 h	Wedelolactone	HPLC	Wedelolactone (13.71 ± 0.82 mg/100 g *E. alba)*	[64]
	Aerial parts	Methanol–water system	SLR of 1:100 of 70% methanol for 180 min	Total phenolics, wedelolactone	HPLC	Wedelolactone yield (4.01 ± 0.08 mg/g), TPC (13.79 ± 0.40 mg/g)	[62]
	Leaves	Ethanol	Freeze–dried sample, SLR of 1:100 (*w*/*v*), 10 h at 80 °C	Total phenolics	LC–ESI–QTOF–MS/MS, HPTLC	TPC (30.7 ± 1.1 mg GAE/g), wedelolactone, luteolin, other phenolic compounds	[22]
	Aerial parts	Methanol, hexane, ethanol, water	SLR of 1:9 of solvent at 50 °C for 36 h	Wedelolactone	TLC, HPLC	Best yield (76% with methanol)	[65]
	Leaves	Methanol	SLR of 1:150 at 90 °C for 6 h	Wedelolactone	HPLC	Wedelolactone yield (5.05 mg/g)	[54]
	Leaves	Methanol	SLR 1:100 at 90 °C for 6 h	Wedelolactone	HPLC	Wedelolactone yield (0.7 mg/g)	[55]
	Whole plants	Methanol	SLR of 1:6 for 12 h	Wedelolactone	HPTLC	Wedelolactone yield (0.48% *w*/*w*)	[53]
Liquid–liquid extraction (Aqueous two-phase extraction)	Leaves	Polyethylene glycol, sodium citrate	SLR of 1:40 using PEG 6000,18% (*w*/*v*), PEG concentration, Sodium citrate salt concentration, 17.96% (*w*/*v*), and pH 7 for 2 h	Wedelolactone	HPLC	Wedelolactone yield (6.52 mg/g)	[54]
Hydrodistillation	Aerial parts	Water	SLR of 1:10, soaked for 12 h, distilled for 3 h	Volatile compounds	GC–MS	Heptadecane, *n*-hexadecanoic acid, pentadecane, and other 52 volatile compounds	[66]
Supercritical carbon dioxide extraction	Whole plants	Liquid carbon dioxide	25 MPa using cosolvent of 9.44% at 56 °C for 60 min	Wedelolactone	HPLC	Wedelolactone (15.37 ± 0.63 mg/100 g *E. alba)*	[64]
	Whole plants	Liquid carbon dioxide	CO_2_ flow rate—23.98 mL/min, Pressure—4000–6000 psi, at 40–50 °C	Wedelolactone	HPTLC	Wedelolactone yield (0.002–0.013% *w*/*w*)	[53]
Ultrasound-assisted extraction	Aerial parts	Ethanol–water system	SLR of 1:50 using 48% of ethanol in water temperature at 40 °C and 90 W for 11 min	Total phenolics, wedelolactone	HPLC	Wedelolactone yield (3.90 ± 0.10 mg/g), TPC (22.57 ± 0.90 mg/g)	[62]
	Aerial parts	Ethanol–water system	SLR of 1:33 of 90% ethanol in water using frequency of 40 kHz for 26.5 min	Wedelolactone	HPLC	Wedelolactone yield (63.16 ± 0.10%)	[60]
	Leaves	Methanol	SLR of 1:60 at 50 °C using 60% duty cycle for 45 min	Wedelolactone	HPLC	Wedelolactone yield (0.62 mg/g)	[55]
	Whole plants	Methanol	SLR of 1:5, for 45 min	Wedelolactone	HPTLC	Wedelolactone yield (0.36% *w*/*w*)	[53]
	Leaves	Ethanol–water system	SLR of 1:14 using 70% of ethanol in water at 70 °C for 3 h	Total saponins	-	Saponins content (2.096%)	[67]
Microwave-assisted extraction	Aerial parts	Ethanol–water system	SLR of 1:33 of 90% ethanol in water at 208 W for 26.5 min	Wedelolactone	HPLC	Wedelolactone yield (82.67 ± 0.16%)	[60]
	Whole plants	Methanol	SLR of 1:5, 100 W for 15 min	Wedelolactone	HPTLC	Wedelolactone yield (0.27% *w*/*w*)	[53]
Ultrahigh pressure-assisted extraction	Aerial parts	Methanol–water system	SLR of 1:20 using 80% methanol in water at 100 MPa pressure for 3 min	Wedelolactone	HPLC	Wedelolactone yield (2.4 mg/g) isodemethylwedelolactone yield (0.7 mg/g)	[63]
Maceration-percolation	Whole plants	Methanol	SLR of 100 g/500 mL for 24 h, followed by percolation until the percolate was colorless	Wedelolactone	HPTLC	Wedelolactone yield (0.38% *w*/*w*)	[53]
Ultrasound and microwave-assisted extraction	Aerial parts	Ethanol–water system	SLR of sample, 80% ethanol in water, and hydrochloric acid:1: 50: 0.3 (*w*/*v*/*v*), microwave power: 40 W, for 9 min (3 min × 3 cycles)	Luteolin	RP–HPLC	Luteolin (0.690 mg/g)	[61]

**Table 3:** Summary of bioactive compound separation and purification techniques used for *E. alba*.

Part of the Plant	Procedure	Operating Parameters			Targeted Compounds	References
		Stationary Phase	Detector	Mobile Phases and Conditions		
Whole plants	HPLC	C18 column	PDA detector at 352 nm	Mobile phase (MP)—methanol: water: acetic acid, 0.6 mL/min, the injection volume (IV)—10 µL	Wedelolactone	[83]
	CC, HPTLC	silica gel CC (60–120 mesh)		Eluted with toluene, HPTLC—a pre-activated silica gel HPTLC plate, mobile phase toluene: ethyl acetate	Wedelolactone	[53]
	HPLC	C-18 column	UV detector at 351 nm	MP—methanol—acetic acid (0.5%) buffer, 0.5 mL/min at 40 °C	Wedelolactone	[64]
Aerial parts	HSCCC	Separation column	UV detector at 254 nm	Petroleum ether–ethyl acetate–methanol–water (3:7:5:5), 20 mL/min for stationary phase (SP), 1.5 mL/min for MP	Wedelolactone, isodemethylwedelolactone, and luteolin	[63]
	HPLC	A Thermo ODS2-Hypersil column	PDA detector at 350 nm	MP—acetonitrile (A) and 0.1% formic acid aqueous solution (B), 1 mL/min, IV—10 µL	Wedelolactone	[62]
	HPLC	C18 reversed-phase column	PDA detector at 352 nm	MP—methanol: 0.4% phosphoric acid, 1 mL/min, IV—10 μL at 30 °C	Luteolin	[61]
	HPLC	C18 column	UV detector at 249 nm	MP—0.5% aqueous glacial acetic acid; 1 mL/min at 30 °C, IV—20 µL	Wedelolactone	[60]
	CC, TLC	Silica gel CC		Methanol and chloroform	Wedelolactone	[65]
	CC, HPLC	1. Sephadex LH-20 CC; 2. Silica gel CC; 3. ODS; 4. Preparative HPLC	G1365D Multiple Wavelength Detector	1. Dichloromethane–water; 2. Methanol; 3. Methanol; 4. Methanol	Ecliptasaponin A, 7-*O*-methylorobol-4′-*O*-β-D-glucopyranoside, 3-oxo-16α-hydroxy-olean-12-en-28-oic acid, 3′-hydroxybiochanin A, echinocystic acid, echinocystic acid 28-*O*-β-D-glucopyranoside, eclalbasaponin I and IV	[82]
	CC, TLC	1.macroporous resin CC; 2. Silica gel CC for fractionation; 3,4. Sephadex LH-20 CC; 5. ODS CC		1. Ethanol–water; 2. Chloroform–methanol–water; 3. Methanol; 4. Chloroform–methanol; 5. Methanol–water	Wedelolactone, Eclalbasaponin I and luteolin, Luteolin-7-*O*-glucoside	[10]
	CC, HPLC	1. D-101 macroporous resin CC; 2. Silica gel CC; 3. Sephadex LH-20 CC; 4. RP-18 CC for subfractions; 5. Silica gel CC; 6. Semipreparative HPLC—Agilent SB-C18 column, ODS-A column for isolation and purification		1. Ethanol–water; 2. Petroleum ether–ethyl acetate; 3. Dichloromethane–methanol; 4. Methanol–water; 5. Dichloromethane–methanol; 6. 65% Acetonitrile–water	Ecliprostins A–C	[11]
	HPLC	5C18-AR-II analytical column	UV detector at 320 nm	MP—10 mm KH_2_PO_4_, pH 4.0 and acetonitrile: methanol: water, 0.8 mL/min, IV—20 µL	Chlorogenic acid	[58]
	CC, HPLC	1. Silica gel CC; 2. ODS CC; 3. Sephadex LH-20 CC; 4. Semipreparative HPLC		1. Dichloromethane–methanol–water; 2.,3., and 4. Methanol–water.	Three new triterpenes	[12]
Leaves	HPLC	C-18 column	DAD detector at 351 nm,	MP—Methanol–water, 0.3 mL/min, at 30 ± 2 °C	Wedelolactone	[54]
	HPLC	C18 column	UV detector at 268 nm	MP—acetonitrile: 1% formic acid, 1 mL/min at 20 °C	Luteolin	[84]
	HPLC	C-18 column	DAD detector at 351 nm,	MP—Methanol–water acidified with 0.1% acetic acid, 1 mL/min	Wedelolactone	[55]

**Table 4:** Summary of bioactive compound identification and characterization techniques used for *E. alba*.

Part of the Plant	Identification and Characterization	Operating Parameters	Bioactive Compounds	References
Whole plants	TLC	SP—silica gel 60, MP—chloroform–methanol, detecting agent—iron (III) chloride	Wedelolactone	[94]
	GC–MS	A GC–MS with an elite column, IV—2 μL with temperature range from 40 to 280 °C, carrier gas—helium, 1 mL/min	Various phytochemical compounds	[95]
Aerial parts	NMR	^1^H–NMR at 400 MHz and ^13^C–NMR at 101 MHz using dimethyl sulfoxide as a solvent	Luteolin, luteolin-7-*O*-glucoside, wedelolactone, and eclalbasaponin I,	[10]
	LC–MS	UHPLC–Q–TOF–MS UPLC—HSS T3 column, MP—acetonitrile and water (containing 0.1% formic acid), IV—5 μL, 0.3 mL/min at 30 °C. LC–QQQ–MS HPLC—C18 column, MP—acetonitrile and water (containing 0.1% formic acid), IV—1 μL, 0.5 mL/min at 35 °C.	Luteolin-7-*O*-β-D-glucopyranoside, luteolin, apigenin, ecliptasaponin A, C, and I,	[96]
			28-*O*-β-D-glucopyranoside, echinocystic acid, and 3-oxo-16α-hydroxy-olean-12-en-28-oic acid	
	GC–MS	HP–5 MS capillary column (5% diphenyl, 95% dimethyl polysiloxane) at 70 eV, 50–600 amu of mass scan range, 1 mL/min, IV—0.1 μL with temperature range from 60 °C to 215 °C for 45 min	Fifty-nine volatile compounds, including α-pinene, caryophyllene, α-humulene, α-pinene, camphene, allo-aromadendrene, α-amorphene	[97]
	GC–MS	GC–HP–5MS capillary column at 70 eV, maximum temperature at 350 °C, helium as carrier gas, 1 mL/min, IV—1 μL	Heptadecane, *n*-hexadecanoic acid, pentadecane, and other 52 volatile compounds	[66]
	LC–MS, NMR	ESI–MS analyses—Triple Quad LC–MS instrument, HR–ESIMS analysis—Q–TOF mass spectrometer, NMR—a Bruker Avance DRX600 spectrometer, 2D ^1^H-^1^H COSY and HMBC NMR	Ecliprostins A–C	[11]
	FT–IR, LC–MS, NMR, UV	Varian 640-IR FT–IR spectrophotometer, Negative-ion HRESI–TOF–MS, Bruker 500 MHz NMR at 500 and 125 MHz, using tetramethylsilane (TMS), UV–Vis spectrometer	Ecliptasaponin A, 7-*O*-methylorobol-4′-*O*-β-D-glucopyranoside, 3-oxo-16α-hydroxy-olean-12-en-28-oic acid, 3′-hydroxybiochanin A, echinocystic acid, echinocystic acid 28-*O*-β-D-glucopyranoside, eclalbasaponin I and IV	[82]
	NMR, MS, IR, UV, GC–MS	1D, 2D NMR spectra and TOF LC–MS, FT–IR spectrometer, UV–Vis spectrophotometer, GC apparatus using an L-Chirasil-Val column	Three new triterpenes	[12]
Leaves	GC–MS	Elite-5MS (5% diphenyl/95% dimethyl polysiloxane), a capillary column, at 70 eV, IV—2 μL with temperature range from 110 to 280 °C, and the carrier gas—helium, 1 mL/min for 36 min	Methyl ester, methyl, methyl ester, pentadecanic aciddiethyl phthalate, glycine, c-sitosterol, eicosyl ester, and 10-octadeconic acid	[98]
	GC–MS	REX column, Temperature range 70–300 °C, helium—carrier gas, IV—2 μL	Glycine, hydrazine carboxyamide, garbamic acid, naphthoquinone, and other substances	[99]
	LC–MS	LC–ESI–QTOF–MS/MS—C18, 500 µL/min, IV—5 µL, MP—water + 0.1% formic acid and acetonitrile + 0.1% formic acid at 35 °C	Phenolic acids (gallic acid, protocatechuic acid, etc.), Flavonoids (luteolin, etc.), wedelolactone, triterpenoids, and phenolic aldehyde (protocatechualdehyde)	[22]
	HPTLC	Silica 60F 254 on aluminum sheet, 10 s/µL of syringe injection rate; IV—2 µL for plant extract and 1 µL for standards; MP—toluene–ethyl acetate–formic acid for 5 min at room temperature	Wedelolactone, luteolin, chlorophyll	[22]
	TLC, Q–TOF–MS, UV, NMR, FT–IR	Silica gel 60 F254 precoated plates, MP—ethyl acetate–toluene–formic acid–methanol, the spraying agent—NP-PEG, Micromass, Q–TOF–MS, UV–Vis spectrophotometer, Bruker AV-500 NMR spectrometer using DMSO as a solvent	Luteolin	[84]
	FT–IR	BRUKER IFS 66 model FT–IR spectrometer	Wedelolactone	[24]
	GC–MS	Elite-5MS (5% diphenyl/95% dimethyl polysiloxane), a capillary column, at 70 eV, IV—2 μL with temperature range from 110 to 280 °C, and the carrier gas—helium, 1 mL/min for 36 min	2-ethyl-2-methyl, 1-Heptatriacotanol, butyl octyl ester, Dodecanoic acid, Oleic acid, eicosyl ester, 9,19- Cyclocholestan-3-ol-7-one,4a-dimethly-[20R], 10- Octadecenoic acid, c-Sitosterol, methyl ester, 1,2 Benzenedicarboxylic acid, 10 methyl, methyl ester, Tridecanol	[100]
	SEM–EDS	20 kV, high vacuum mode	Elements including sodium, magnesium, potassium, calcium, copper, zinc, and iron	[24]
Stems	FT–IR	BRUKER IFS 66 model FT–IR spectrometer	Wedelolactone	[24]
	SEM–EDS	20 kV, high vacuum mode	Elements including sodium, magnesium, potassium, calcium, copper, zinc, and iron	[24]
Roots	SEM–EDS	20 kV, high vacuum mode	Elements including sodium, magnesium, potassium, calcium, copper, zinc, and iron	[24]
	FT–IR	BRUKER IFS 66 model FT–IR spectrometer	Wedelolactone	[24]

## Data Availability

Not applicable.

## Abbreviations

ATPE: Aqueous two-phase extraction; UAE: Ultrasound-assisted extraction; MAE: Microwave-assisted extraction; SFE: Supercritical fluid extraction; UHPE: Ultrahigh pressure-assisted extraction; HD: Hydrodistillation; CC: Column chromatography; ODS: Octadecyl-silica; HPLC: High-performance liquid chromatography; HSCCC: High-speed counter-current chromatography; TLC: Thin-layer chromatography; CCD: Central composite design; RSM: Response surface methodology; HPTLC: High-performance thin-layer chromatography; UV–Vis: Ultraviolet-visible; FT–IR: Fourier-transform infrared spectroscopy; SEM–EDS: Scanning electron microscopy with energy-dispersive X-ray spectroscopy; NMR: Nuclear magnetic resonance; COSY: Correlation spectroscopy; HMBC: Heteronuclear multiple-bond correlation spectroscopy; LC–MS: Liquid chromatography–mass spectrometry; GC–MS: Gas chromatography–mass spectrometry; PDA: Photodiode array; TPC: Total phenolic content; TFC: Total flavonoid content; GAE: Gallic acid equivalence; SLR: Solid–liquid ratio; MP: Mobile phase; SP: Stationary phase; IV: Injection volume.
